# Draft Genome Sequence of an Endophytic Biocontrol Bacterium, Bacillus velezensis PG12, Isolated from Apple Fruit

**DOI:** 10.1128/MRA.00468-19

**Published:** 2019-10-10

**Authors:** Qingchao Zeng, Jianbo Xie, Yan Li, Xinyi Chen, Qi Wang

**Affiliations:** a College of Plant Protection, Key Laboratory of Plant Pathology, Ministry of Agriculture, China Agricultural University, Beijing, China; b College of Biological Sciences and Technology, Key Laboratory of Genetics and Breeding in Forest Trees and Ornamental Plants, Ministry of Education, Beijing Forestry University, Beijing, China; University of Maryland School of Medicine

## Abstract

Bacillus velezensis PG12 is a biocontrol endophytic bacterium that is capable of inhibition of a broad range of plant-pathogenic fungi. The strain has the potential to be developed into a biocontrol agent for use in agriculture. Here, we report the draft genome sequence of *B. velezensis* PG12, which contains 22 scaffolds (3,990,845 bp), 3,884 coding sequences (CDSs), and an average G+C content of 46.45%.

## ANNOUNCEMENT

Bacillus velezensis is a heterotypic synonym of Bacillus methylotrophicus, Bacillus amyloliquefaciens subsp. *plantarum*, and Bacillus oryzicola, based on comparative genomics and DNA-DNA relatedness calculation (1). The members of the genus Bacillus are ubiquitous in nature and include biologically and ecologically diverse species that range from those beneficial to economically important plants to pathogenic species that are harmful to humans (2). *Bacillus* species, as important biopesticides, provide a wealth of resources for biocontrol, and strains belonging to this genus are known to produce a diverse spectrum of secondary metabolites with antimicrobial activity (3). *B. velezensis*, which is a Gram-positive, rod-shaped bacterium belonging to the class Bacilli, has been widely used as a biological control agent in agricultural fields due to its strong ability to suppress plant-pathogenic fungi (4, 5). For these reasons, Bacillus species are good candidates for use as biofertilizers or biopesticides to improve crop yield and quality. Meanwhile, some of them have already been commercialized for increasing crop yield (3, 5).

*B. velezensis* PG12 is an endophytic bacterium that was isolated from apple fruit in northern China. This strain shows a strong antagonistic activity against apple ring rot. In addition, strain PG12 suppresses a broad spectrum of pathogenic fungi (6). Here, we report the draft genome sequence of *B. velezensis* PG12 and its annotation in order to facilitate its application in the biocontrol of plant diseases.

*B. velezensis* PG12 was routinely grown at 37°C on Luria-Bertani (LB) broth or on solid LB medium supplemented with 1.5% agar (6). Strain PG12 was cultured for 12 h at 180 rpm and 37°C, and the total genomic DNA of *B. velezensis* PG12 was extracted using the phenol-chloroform method (7). The quantity and quality of isolated DNA were determined using a NanoDrop spectrophotometer. Then, DNA was sequenced using the Illumina HiSeq 2500 platform at Berry Genomics Bioinformatics Technology Co., Ltd. (Beijing, China). Two libraries were constructed for sequencing. First, genomic DNA was fragmented, and inserts of 350 bp were selected to construct a paired-end indexed library. Second, genomic DNA was fragmented, and inserts of 2 kb were selected to construct a mate-paired indexed library. For the library construction, the TruSeq Nano DNA sample preparation kit and mate pair library prep kit v2 were used following the manufacturer’s instructions. Data processing was done with default parameters. Adapters and low-quality sequences were removed using Cutadapt (version 1.18) and Sickle (version 1.33) software, respectively (8, 9). Finally, we obtained 1.38 Gb of data (350-bp library) and 1.24 Gb of data (2-kb library) after data trimming. The genome coverage was 671×. The high-quality reads were then assembled using SOAPdenovo software (version 2.04) with a kmer value of 81 (10). Gaps between scaffolds were closed using GapCloser (version 1.12) with the default setting (10). Annotation was performed using Prokka software (version 1.11) using default parameters (11). Putative proteins were searched against the Clusters of Orthologous Groups (COG), NCBI nonredundant (NR) protein, Gene Ontology (GO), and Kyoto Encyclopedia of Genes and Genomes (KEGG) databases.

The high-quality draft genome of *B. velezensis* PG12 was distributed in 22 scaffolds with a total size of 3,990,845 bp, an *N*_50_ value of 2,085,242 bp, and an average G+C content of 46.45%. Genome analysis showed that the genome of strain PG12 contained 3,884 protein coding genes (coding sequences [CDSs]), 8 rRNAs, and 56 tRNAs. The predicted protein coding genes represented 89.53% of the total genome sequence and had a total length of 3,572,903 bp. The average nucleotide identity was calculated using JSpecies software (version 1.2.1) (12). The genome of strain PG12 was found to be closely related to that of *B. velezensis* CAU B946, with an average nucleotide identity of 99.96%. AntiSMASH (version 3.0) online prediction software using default parameters showed that several genes, including iturin, fengycin, and surfactin biosynthesis genes, were represented in the genome sequence of *B. velezensis* PG12.

The genome sequence of *B. velezensis* PG12 and its genome annotation provide deeper insights into understanding the molecular genetic characteristics of *B. velezensis* and further understanding of the molecular mechanism for controlling apple ring rot, which is beneficial for the development of microbial fertilizers or biocontrol agents to improve crop production.

### Data availability.

This whole-genome shotgun project has been deposited at DDBJ/ENA/GenBank under the accession number PIWI00000000 (assembly accession number GCF_002835205). The version described in this paper is the first version, PIWI01000000 (assembly accession number GCF_002835205.1). The raw data have been registered in the NCBI SRA database under the accession numbers SRR8935607 and SRR8935608.
